# *Mycoplasma penetrans* bacteremia in an immunocompromised patient detected by metagenomic sequencing: a case report

**DOI:** 10.1186/s12879-019-4723-7

**Published:** 2020-01-03

**Authors:** Benjamin Preiswerk, Frank Imkamp, Denise Vorburger, Rico V. Hömke, Peter M. Keller, Karoline Wagner

**Affiliations:** 1 Institute of Medical Microbiology, University of Zurich, Zurich, Switzerland; 2 Present Address: Triemli Hospital Zurich, Zurich, Switzerland; 3 Department of Gynecology, University Hospital Zurich, Zurich, Switzerland; 4 Present Address: Institute for Infectious Diseases, University of Bern, Bern, Switzerland; 5 Laboratory Medicine, University Hospital of Basel, Petersgraben 4, 4031 Basel, Switzerland

**Keywords:** *Mycoplasma penetrans*, Bacteremia, Immune deficiency, Metagenomic sequencing

## Abstract

**Background:**

*Mycoplasma* sp. are well recognized as etiological agents of respiratory and sexually transmitted disease. *Mycoplasma penetrans,* a species of *Mycoplasma* sp., has been frequently detected in HIV-positive patients and associated with the progression of HIV-associated disease. To date, there is only a single case report describing *M. penetrans* as the causative agent of a severe respiratory tract infection in a HIV-negative patient.

**Case presentation:**

In this report, we describe the case of *M. penetrans* bacteremia in a HIV-negative, 38-year-old, female, immunocompromised, solid organ transplant patient (combined kidney and pancreas transplantation in 2016)*,* who was admitted to our hospital with anemic uterine bleeding and fever of 38.3 °C. Several hours before her admission at our university hospital, a latex bladder catheter was inserted into her uterus and she complained about fatigue, dizziness and ongoing vaginal bleeding. Laboratory examination showed severe anemia, but microbiological examination was inconspicuous (culture negative vaginal and cervical smears, negative urine culture). Bacterial blood cultures showed a growth signal after 4 h, but microscopic examination with Gram staining and subcultures on different agar media did not identify bacterial pathogens. To identify the bacterial cause of malignancy in the patient, metagenomic sequencing of the blood culture was performed that identified *M. penetrans.*

**Conclusion:**

Metagenomic sequencing identified *M. penetrans* in an immunosuppressed patient with culture-negative bacteremia. Clinicians should be aware of the opportunistic potential of *M. penetrans* that may cause severe infections in certain vulnerable patient populations and the limitations of culture and Gram staining for confirming the presence of fastidious bacterial pathogens like *Mycoplasma* spp.

## Background

*Mycoplasmataceae* are among the smallest self-replicating organisms known and the only described prokaryotes that lack a cell wall [1]. They have an extremely reduced genome size of around 1 Mb and lack most genes required for nutrient metabolism and therefore often adopt a parasitic lifestyle in their host organisms. *Mycoplasmataceae* have been isolated from various body sites in humans; however, only a few species have been well recognized as etiological agents of disease [2–8]. Among these the most prevalent pathogenic *Mycoplasma* spp. in humans are *Mycoplasma pneumoniae* that causes respiratory tract infections and pneumonia, and the widespread sexually transmitted *Mycoplasma genitalium*. *Mycoplasma hominis* and the closely related species *Ureaplasma urealyticum* are common colonizers of the urogenital tract and have been associated with chronic urogenital infections [9, 10].

For the detection of bacterial pathogens in patients with bacteremia, blood culture is still the most commonly used diagnostic method in the bacteriology laboratory. However, culture and Gram staining often remain negative when fastidious, cell-wall free microorganisms such as *Mycoplasma* spp. are the causative agent of infection. Therefore, rapid detection of microorganisms is pivotal for patient management and initiation of adequate antimicrobial therapy. In cases in which blood culture remains negative but the clinical signs and symptoms of the patient strongly indicates infectious disease, diagnosis can be achieved by using metagenomic sequencing. Metagenomics allows culture-independent sequencing of the pathogen’s genome directly from the clinical specimens and potentially provides insights into the pathogen’s virulence (by detection of virulence genes) and drug susceptibility (by identification of resistance mutations in target genes).

## Case presentation

**Fig. 1:**
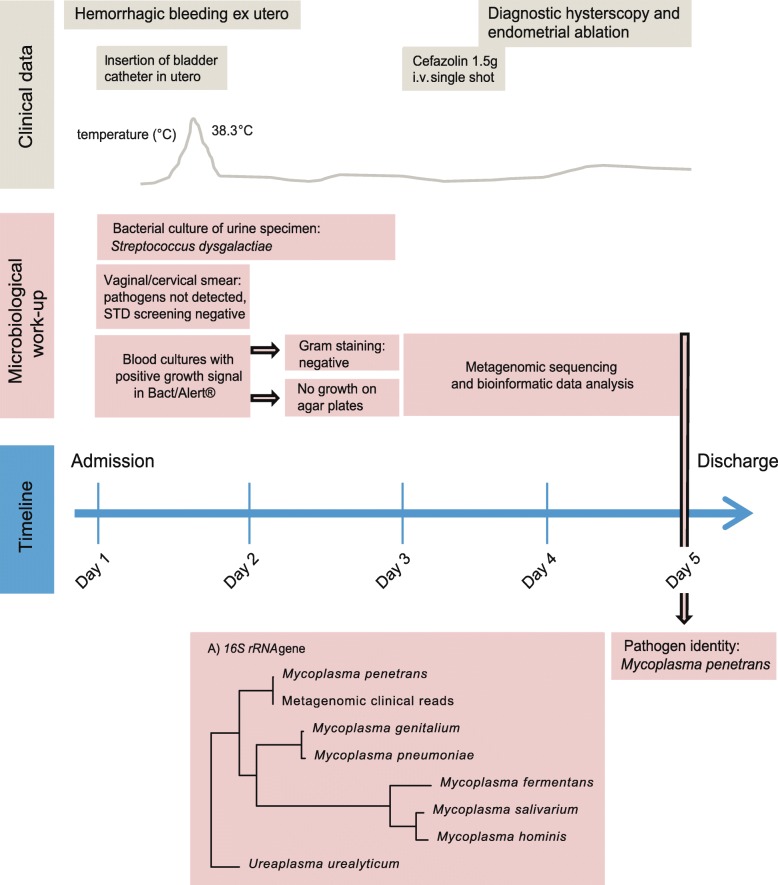
Clinical data, microbiological work-up of samples and timeline of diagnostic testing and clinical procedures

In June 2018, a 38-year-old female patient was admitted to our hospital with anemic uterine bleeding and fever of 38.3 °C (Fig. 1). Her personal history was remarkable for diabetes mellitus type I, a combined kidney and pancreas transplantation in 2016 and known uterine myomas, which were the origin of several episodes of hemorrhagic bleeding within the last 2 years. The nulliparous patient was in regular gynecological examination without surgical interventions. She was denied implantation of any levonorgestrel containing intrauterine device because of concerns about elevated risk of foreign-body associated infection in her immunocompromised state (dual immunosuppression with tacrolimus and mycophenolate mofetil; both with stable drug levels).

Upon admission at our university hospital, the patient complained about fatigue, dizziness and ongoing vaginal bleeding. Several hours before admission, a latex bladder catheter was inserted into her uterus, and she received an intravenous hemostatic treatment with tranexamic acid and fibrinogen in another hospital. On examination, the patient was in a reduced general condition but alert and fully oriented. The temperature was 38.3 °C, the blood pressure 107/45 mmHg, the pulse 90 beats per minute, and the respiratory rate and oxygen saturation were in normal range.

Laboratory examination showed severe anemia (hemoglobin 40 g/L, hematocrit 0.137 L/L, erythrocyte count [1.37 × 10^12^/L]) but normal leucocyte (4.38 × 10^9^/L) and thrombocyte counts (193 × 10^9^/L). C-reactive protein was in a normal range (3.3 mg/L).

A microbiological diagnostic workup was undertaken. The vaginal smear grew normal flora and *Staphylococcus aureus*. The cervical smear was PCR-negative for *Neisseria gonorrhoeae* and *Chlamydia trachomatis.* Two out of four blood culture bottles turned positive after 5 h (Bact/Alert® Virtuo, BioMérieux, Marcy-l’Etoile, France). However, microscopy with Gram stain showed no microorganisms, and subcultures on different agar media (i.e. Columbia sheep blood agar, colistin-nalidixic acid agar, chocolate agar, MacConkey agar, brucella agar, phenylethyl alcohol agar [all BioMérieux] and a modified Shepard agar medium [A7; ELITech, Puteaux, France] remained without growth. In the effort to identify the cause of the positive growth signal in the blood culture, 5 ml of the blood culture was used for metagenomic sequencing on an Illumina MiSeq platform*.* In detail, 2 ml erythrocyte lysis buffer (Qiagen, Hilden, Germany) was added to 5 ml of negative blood culture. It was vortexed at maximum speed for 15 s and afterwards incubated for 5 min at room temperature, followed by addition of 0.1 volume of 10X Turbo DNAse buffer and 3 μl of Turbo DNAse (Thermo Fischer Scientific Inc.). The sample was gently mixed and incubated at 37 °C for 30 min. Afterwards, it was centrifuged at maximum speed for 10 min, and the supernatant was removed. The pellet was resuspended in 650 μl of pre-warmed PM1 buffer from the AllPrep® PowerFecal® DNA/RNA Kit (Qiagen). DNA extraction from bacterial cells was performed according to the manufactures’ instructions. Library preparation was done using the Qiagen® QIAseq FX DNA Library Kit (Qiagen, Hilden, Germany), according to the producers’ recommendations. Sequencing library quality and size distribution were analyzed on a Fragment analyzer automated CE system (Advanced Analytical Technologies Inc., Heidelberg, Germany), according to the manufacturer’s instructions using the Fragment Analyzer 474 HS NGS Fragment Kit. Sequencing libraries were paired-end sequenced (2 × 150 bp) on an Illumina MiSeq platform (Illumina®, San Diego CA, USA).

**Fig. 2:**
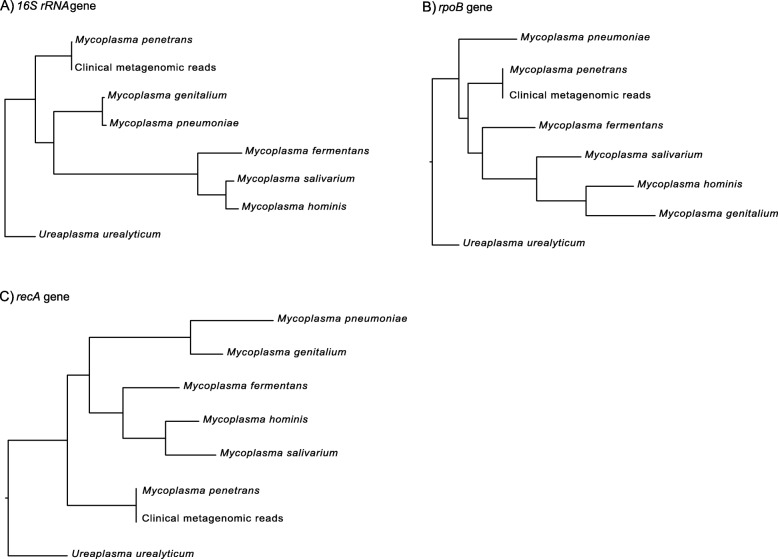
Phylogenetic analysis of clinical metagenomic reads detected by metagenomic sequencing of the negative blood culture from the patient with a growth signal in the BacT/ALERT system (i.e. clinical metagenomic reads). Phylogenetic trees were constructed using the full length 16S rRNA (**a**), the *rpoB* (**b**) and the *recA* gene sequences (**c**) of different *Mycoplasma* sp. with *Ureaplasma urealyticum* as outlier

Raw sequencing reads (FASTQ) were filtered and trimmed using TRIMMOMATIC [11], applying a threshold PHRED score of 25. Phylogenetic analysis was done by using the full length 16S rRNA, *rpoB* and *recA* gene sequences of the following *Mycoplasma* spp.: *M. penetrans* HF-2 (NCBI accession number: BA000026.2), *M. genitalium* G37 (NCBI accession number: NC_000908.2), *M. pneumoniae* M129 (NCBI accession number: NC_000912.1), *Mycoplasma fermentans* M64 (NCBI accession number: NC_014921.1), *Mycoplasma salivarium* ATCC_23064 (NCBI accession number: NZ_AXZE01000009.1), *M. hominis* ATCC_23114 (NCBI accession number: NC_013511.1), and as outlier *U. urealyticum* serovar 10 ATCC 33699 (NCBI accession number: NC_011374.1) was included in the phylogenetic analysis. Phylogenetic trees were generated with the FastTree MP software [12] (bootstrap 1000 option, maximum likelihood GTR [generalized time-reversible model] option). The metagenomic sequence reads covered 99.2% of the published reference sequence of 1.35862 Mb [6]. In the 16S rRNA gene, two mismatches were found at nucleotide positions C98T and T221C compared to the reference strain sequence. This combined genetic information points towards the presence of *M. penetrans* in the blood cultures (Fig. 2). In order to confirm the phylogenetic analysis, metagenomic sequences of the negative blood culture from the patient with a growth signal in the BacT/ALERT system were classified using Kaiju [13] and yielded 363′851 reads, of which 78% were annotated as *M. penetrans* (Additional file 2: Table S1, Additional file 1: Figure S1). An inoculated blood culture without a growth signal in the BacT/ALERT system and the non-inoculated blood culture growth medium were sequenced as negative controls for the meta-genomic sequencing approach (Additional file 3: Table S2, Additional file 4: Table S3). While no *M. penetrans* reads were identified in both negative controls, the most frequently detected reads assigned to *Enterococcus* sp., *Streptococcus* sp. and *Staphylococcus* sp. (Additional file 1: Figure S1).

The patient was taken to the operating theatre for diagnostic hysteroscopy and endometrial ablation. She received a preoperative single shot dose of cefazolin (2 g) intravenously. Since the postoperative course was without complications, the patient remained afebrile and the uterine bleeding stopped after the removal of intrauterine catheter. The *M. penetrans* bacteremia was self-limiting, requiring no further antibiotic treatment, and the patient was discharged in stable general condition.

## Discussion

In this study, we report the case of an immunocompromised 38-year-old female patient with culture negative bacteremia that most likely results from a preceding urogenital *M. penetrans* colonization*.* Previously, only a few studies have focused on bacteremia caused by urogenital *Mycoplasma* spp. [14, 15]. Colonization rate of *M. penetrans* in the urogenital tract of healthy women and men is unknown; however, *M. penetrans* seroprevalence was < 1% in HIV-negative blood donors [16]. In congruence, other studies confirmed a low *M. penetrans* seroprevalence of 0.3% in the general population [17]. In contrast, high *M. penetrans* seroprevalence was found in HIV-positive patients and increased with progression of HIV-associated disease [2, 17, 18]. Taken together, these findings indicate that *M. penetrans* seroprevalence may be associated with the immune status of the patient. However, reports on *M. penetrans* infection in HIV-negative individuals are scarce in the literature. To date, only a single case report exists describing the isolation of *M. penetrans* from a HIV-negative patient with a primary antiphospholipid syndrome, a multisystem autoimmune condition [19].

As there are substantial difficulties in detecting fastidious bacterial pathogens like *M. penetrans* with culture and Gram staining, we believe that there might be a significant “under-detection” of *M. penetrans* colonization and infection. Therefore, more rapid, culture-independent molecular methods are required to circumvent this potential detection bias. In recent years, metagenomic sequencing has proven useful in investigating the pathogenic potential of fastidious microorganisms that can just be laboriously cultivated directly from clinical specimens [20, 21]. However, use of appropriate controls is pivotal as the presence of contaminating DNA in blood cultures, extraction chemicals or sequencing reagents may lead to false interpretation of PCR-based (e.g. 16S rRNA sequencing) or meta-genomic sequencing results. One limitation of meta-genomic sequencing approaches are the still long turn-around times, and thus, meta-genomic sequencing results are often not timely available to the treating physician. This results from the fact that meta-genomic sequencing is mostly just employed when the “first-line of diagnostics” (e.g. Gram stain and culture on solid agar media) has failed. Also in this case report, meta-genomic sequencing results could just be made available to the physician after empiric cephalosporin-based therapy had been administered to the patient and the hysterectomy had been performed. Luckily, the patient cleared the bacteremia spontaneously most probably due to bacterial load reduction via hysteroscopy and improvement of her general condition, making specific antibiotic treatment (e.g. with a macrolide antibiotic) unnecessary. Thus, diagnostic workflows must be improved in order to timely provide meta-genomic sequencing results. This requires more rapid library preparation and sequencing protocols, and most of all, cheaper sequencing chemicals that enable a cost-efficient use of meta-genomic sequencing as “first-line diagnostics” in certain patient populations (e.g. critically ill patients, transplant recipients, immunocompromised patients).

## Conclusion

In conclusion, the patient described in our case report is HIV-negative but immunocompromised (solid organ transplant). To our knowledge, this is the first report of *M. penetrans* bacteremia in an immunocompromised patient*.* Although we cannot track back the route of infection in the patient, we assume that the source of the bacteremia with *M. penetrans* was due to mucosal translocation in the utero-cervical compartment in the context of several gynecological interventions. We could show that metagenomic sequencing directly from clinical specimens in patients with culture negative bacteremia of unknown origin can be a helpful tool to receive an accurate microbiological diagnosis, thus allowing to potentially switch to a targeted antibiotic treatment and stop unnecessary empiric treatment for the purposes of antibiotic stewardship.

## Supplementary information

**Additional file 1: Figure S1.** (A) Metagenomic sequencing of the negative blood culture from the patient with a growth signal in the BacT/ALERT system (363′851 classified reads), (B) of an inoculated blood culture without a growth signal in the BacT/ALERT system (12′500 classified reads) and (C) of the non-inoculated blood culture growth medium (16′047 classified reads).

**Additional file 2: Table S1.** Metagenomic sequencing of the negative blood culture from the patient with a growth signal in the BacT/ALERT system.

**Additional file 3: Table S2.** Metagenomic sequencing of an inoculated blood culture without a growth signal in the BacT/ALERT system.

**Additional file 4: Table S3.** Metagenomic sequencing of the non-inoculated blood culture growth medium.

## Data Availability

Whole genome sequence of the analyzed clinical *Mycoplasma penetrans* isolate is available on NCBI under accession number: RCHY00000000 (BioSample: SAMN10180533).
