# Supplementary figures and images for: Mycoplasma penetrans bacteremia in an immunocompromised patient detected by metagenomic sequencing: a case report
Source: BMC Infect Dis. 2020 Jan 3;20:7. doi: 10.1186/s12879-019-4723-7 (PMC6942334; doi:10.1186/s12879-019-4723-7)

A

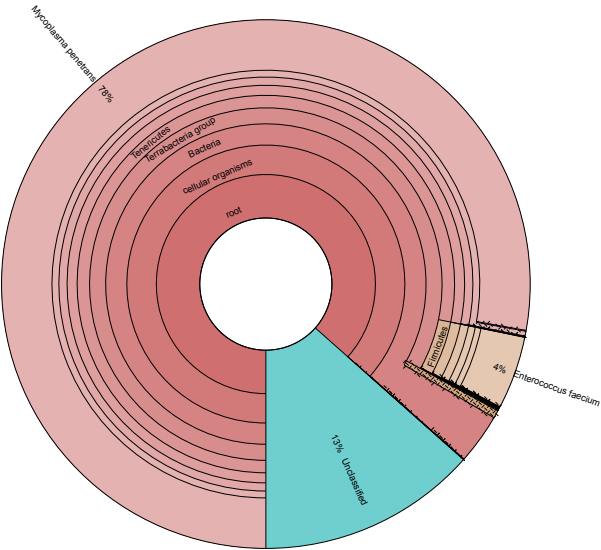

B

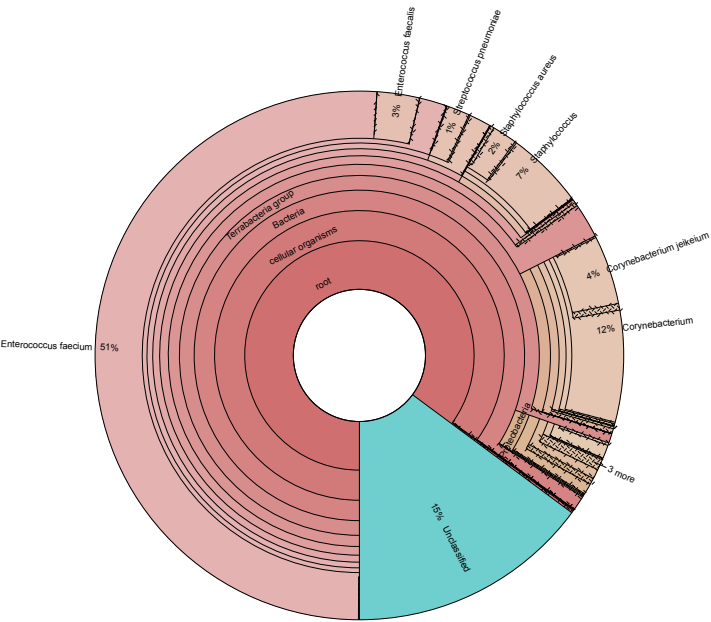

C

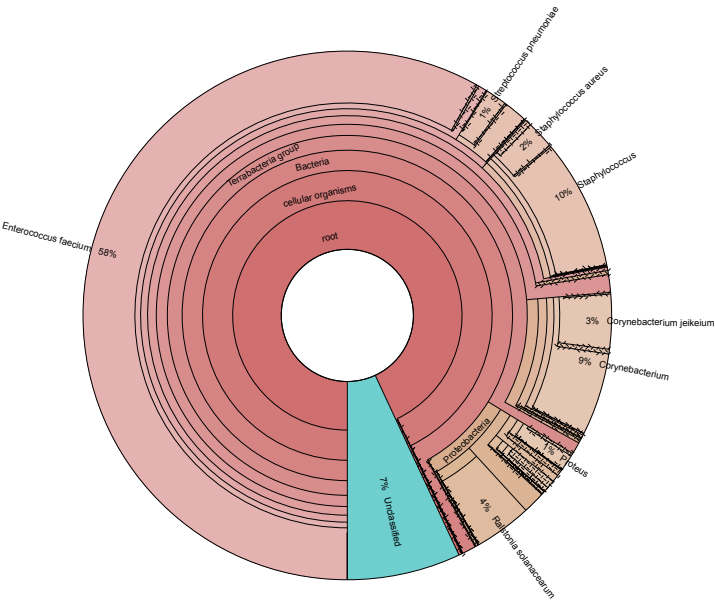

Supplement: Supplementary file 1 — Additional file 1: Figure S1. (A) Metagenomic sequencing of the negative blood culture from the patient with a growth signal in the BacT/ALERT system (363′851 classified reads), (B) of an inoculated blood culture without a growth signal in the BacT/ALERT system (12′500 classified reads) and (C) of the non-inoculated blood culture growth medium (16′047 classified reads). [file 12879_2019_4723_MOESM1_ESM.pdf]
